# Cyclohexane, naphthalene, and diesel fuel increase oxidative stress, *CYP153*, *sodA,* and *recA* gene expression in *Rhodococcus erythropolis*

**DOI:** 10.1002/mbo3.855

**Published:** 2019-05-22

**Authors:** Ivan Sazykin, Maksim Makarenko, Ludmila Khmelevtsova, Ekaterina Seliverstova, Alexander Rakin, Marina Sazykina

**Affiliations:** ^1^ Southern Federal University Rostov‐on‐Don Russian Federation; ^2^ Institute for Bacterial Infections and Zoonoses Friedrich‐Loeffler‐Institut, Federal Research Institute for Animal Health Jena Germany

**Keywords:** cytochrome P450, DNA repair, hydrocarbons, ROS, superoxide dismutase

## Abstract

In this study, we compared the expression of *CYP153*, *sodA*, *sodC,* and *recA* genes and ROS generation in hydrocarbon‐degrading *Rhodococcus erythropolis* in the presence of cyclohexane, naphthalene, and diesel fuel. The expression of cytochrome P450, *sodA* (encoding Fe/Mn superoxide dismutase), *recA,* and superoxide anion radical generation rate increased after the addition of all studied hydrocarbons. The peak of *CYP153*, *sodA,* and *recA* gene expression was registered in the presence of naphthalene. The same substrate upregulated the Cu/Zn superoxide dismutase gene,* sodC*. Cyclohexane generated the highest level of superoxide anion radical production. Hydrogen peroxide accumulated in the medium enriched with diesel fuel. Taken together, hydrocarbon biotransformation leads to oxidative stress and upregulation of antioxidant enzymes and *CYP153* genes, and increases DNA reparation levels in *R. erythropolis* cells.

## Graphical abstract

Under the influence of hydrocarbon, *CYP153*gene expression is amplified, superoxide anion radical generation increases and, as a result, *sodA* and *recA* expression levels increase in *Rhodococcus erythropolis* cells. At incubation with diesel fuel, hydrogen peroxide accumulates in culture medium.

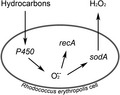

## 1 INTRODUCTION

Hydrocarbon‐ and xenobiotic‐degrading bacteria display enormous plasticity against utilized substrates. These microorganisms are capable of transforming various types of hydrocarbons, their halogen and nitro derivates, pesticides, plastics such as PET and polyethylene, etc. (Danso et al., 2018; Kang et al., 2007; Kato, Miyanaga, Kanaya, & Morikawa, 2009; Lee, 1999; Pérez‐Pantoja, Nikel, Chavarria, & Lorenzo, 2013; Ponce, Latorre, González, & Seeger, 2011; Skariyachan et al., 2017; Sutherland, Horne, Harcourt, Russell, & Oakeshott, 2002; Tamburro et al., 2004). Bacterial cells suffer from an oxidative stress, utilizing such compounds.

Bacteria are unlikely to possess multiple substrate‐specific enzyme systems for oxidation of each compound. For example, bacteria constantly contact oil in natural conditions. Oil is a natural mix composed of more than 1,500 separate substances. Emerging of xenobiotic oxidases is even more uncommon, taking into account that xenobiotics are chemical compounds foreign to bacteria with no history of previous contacts.

A large number of hydrocarbon oxidases lack strict substrate specificity. Enzyme usually oxidizes not only a particular substrate, but a group of substances similar in structure. These substrates can be divided into optimal and suboptimal. Oxidizing suboptimal substrates, these enzymes generate ROS as a result of high‐frequency fault reactions (Lee, 1999; Pérez‐Pantoja et al., 2013; Tamburro et al., 2004). ROS produced by oxygenases leads to oxidative stress and increased mutagenesis in bacterial cells. Interestingly, the transitional forms of enzyme are highly homological to the ancestral form, but acquire expanded substrate specificity and capability to effectively oxidize new substrates (Pérez‐Pantoja et al., 2013). This process facilitates emergence of bacteria with xenobiotic‐oxidizing enzymes. Whether the processes detrimental for a single cell can be beneficial for a bacterial population and whether the oxidative stress can be an adaptive mechanism to increase nutritional substrate variety? The aim of this study was to evaluate the expression of genes, cytochrome P450, superoxide dismutases A and C, *recA*, as well as generation of superoxide anion radical and hydrogen peroxide under the influence of hydrocarbons on *Rhodococcus*.

## 2 MATERIALS AND METHODS

### 2.1 Cultivation of microorganisms

Hydrocarbon‐degrading *Rhodococcus erythropolis* was isolated from a technogenically polluted soil containing polycyclic aromatic hydrocarbons. The isolate was identified by mass spectrometry and 16S rRNA gene sequencing. Mass spectra of ribosomal proteins were obtained using a mass spectrometer MALDI Biotyper (Bruker Daltonik, Germany). Protein profile spectra were imported into the Biotyper program and identified according to standard settings. DNA isolated from this strain was used in the PCR reaction with standard primers for amplification of 16S rRNA: 27 F—AGAGTTTGATCMTGGCTCAG; 1492 R—CGGTTACCTTGTTACGACTT. Sequencing of amplification products was performed by the Sanger method on the ABI 3730 DNA Analyzer (Life Technologies, USA). Sequencing results were processed using the GenBank Blast program (http://blast.ncbi.nlm.nih.gov). The 16S rRNA gene sequence of *R. erythropolis* strain was deposited in the GenBank database under the accession number MH718753.

Bacteria cultivated in Luria‐Bertani (LB) and basic mineral salt medium described before (Sazykin et al., 2016). Fifty‐microliter Erlenmeyer flasks containing 20 ml of medium were cultivated at 170 rpm and 30°C in an orbital shaker incubator Innova 40R (New Brunswick, USA).

Such hydrocarbons as cyclohexane, naphthalene (analytical grade, “Aquatest,” Russia), and commercial diesel fuel were used in the experiments as an additional carbon source.

### 2.2 Superoxide anion radical generation assay

For superoxide anion radical assay, microorganisms were grown overnight (18 hr) in the basic mineral salt medium complemented of 0.5% of yeast extract and 0.5% of tryptone. Suspension of microorganism was triply washed and diluted with basic mineral salt medium to the concentration of 1 × 10^8^ cells per ml. Hundred microliters of culture suspension, 80 μl of basic mineral salt medium, 10 μl of 4 mM deionized water solution of lucigenin (Sigma‐Aldrich, USA), and 10 μl of the hydrocarbon were added to each well of 96‐well microplate COSTAR 3632 (USA).The control sample contained 100 μl of the suspension culture, 90 μl of basic mineral salt medium with the addition of 1% of glucose and 10 μl of 4 mM solution of lucigenin in deionized water.

The plate was incubated for 24 hr in the Luminoskan Ascent microplate luminometer (Thermo Scientific, USA) at 30°C with simultaneous chemiluminescence (CL) measurement every 30 min (48 measurements in total) (Sazykin et al., 2016, 2018). Three independent experiments were carried out and repeated 8 times.

### 2.3 Hydrogen peroxide generation assay

Bacteria were cultivated in 20 ml of basic mineral salt medium with the addition of 2% (400 μl) of hydrocarbons. Basic mineral salt medium with the addition of 2% of glucose was used as the control. The suspension of bacterial cells in the medium before incubation was diluted to the concentration of 1 × 10^6^ cells per ml. Microorganisms were incubated in an orbital incubator for 30 days.

Cultural liquid samples were taken from a flask and centrifuged for 5 min at 14,100 g. For hydrogen peroxide assay, 60 µl of the supernatant of culture medium, 100 µl of PBS, and 20 µl of luminol solution were introduced into a well of a plate. CL measurements were carried out 8 times using a Luminoscan Ascent microplate luminometer. The luminescence of each well was measured within 100 s with the interval of 1 s. Twenty microliters of horseradish peroxidase solution (0.01 u/µl) were added to each plate by means of a built‐in dispenser immediately after the beginning of measurement.

Luminescence level in a well was determined within 100 s after addition of peroxidase with an interval of 1 s. For each measurement, the average value of CL intensity was calculated and subsequently, the biggest average CL value was used (Sazykin et al., 2016, 2018). Three independent experiments were carried out 8 times.

### 2.4 Expression of *CYP153*, *recA*, *sodA*, and *sodC* genes

Bacteria were cultivated in a basic mineral salt medium with the addition 0.5% yeast extract. Approximately 2% w/v hydrocarbons were added to the medium. Basic mineral salt medium with the addition of 0.5% yeast extract and 2% of glucose was used as a control. Strains were cultivated to the late logarithmic growth phase and cells were pelleted at 4,000 g for 2 min.

#### 2.4.1 RNA extraction and cDNA synthesis

RNA was isolated from 25 mg of sample (app. 10^9^ CFU). Eight biological replicates were used for each sample. Samples were thoroughly homogenized with mortar and pestle in the presence of liquid nitrogen.

Total RNA extraction was performed with Extract RNA kit (Evrogen, Russia), based on acid guanidinium thiocyanate–phenol–chloroform extraction method (Chomczynski & Sacchi, 2006). RNA quality and concentration were measured using the NanoDrop 2000 spectrophotometer (Thermo Fisher Scientific, USA) and the Qubit fluorimeter (Invitrogen, USA). All samples had the A260/280 ratio >1.8, ranging between 1.9 and 2.05, as well as no signs of significant ethanol carryover. The concentration of RNA samples was 75–200 ng/μl.

From each sample, 0.5 μg of total RNA was treated with DNAse I (Thermo Fisher Scientific, USA) according to the manufacturer's instruction and DNA‐free RNA was used for further manipulations.

First‐strand cDNA was synthesized using MMLV RT kit (Evrogen, Russia) with random primers. For each sample, we also included a negative control—the same cDNA reaction mix, including RNA, except MMLV.

#### 2.4.2 Quantitative PCR

The qPCR was performed with the designed primers (Table 1) and hot start PCR kit with EvaGreen dye (Syntol, Russia) using the CFX96 Real‐Time PCR Detection System (Bio‐Rad, USA). Each sample was analyzed in triplicate qPCR reactions. The reaction parameters were as follows: 94°C for 5 min (the polymerase activation step); 35 cycles of 94°C for 15 s, 60°C for 20 s, 72°C for 30 s, and 72°C for 5 min (the final elongation step) followed by a melting analysis (0.5°C increment from 60 to 95°C; 10 s per cycle). We designed primers using Primer‐BLAST tool (https://www.ncbi.nlm.nih.gov/tools/primer-blast). The nucleotide sequences of the studied genes were received from the NCBI database. Reaction specificity was controlled using the melting curve analysis and 1.5% agarose gel electrophoresis for each primer pair. No abnormal products were detected.

**Table 1:** Primers designed for the gene expression analysis

Gene	Forward primer sequence (5′−3′)	Reverse primer sequence (5′−3′)	Amplicon size (bp)
*gyrA*	GTCGACGGTCAGGGAAACTT	CGTCGTAGTTCGGGGTGAAA	145
*map*	CATCGAGTCCTACGCCCATC	TCGATCGTGAAGACCATGCC	156
*CYP153*	GTCACGACTGTCCCAATGGT	TCACTGCGTACAACCACGAA	143
*recA*	GAGATCGAAGGCGAGATGGG	TTCTCGCGAAGCTGGTTGAT	134
*rpoB*	GACGACATCGACCACTTCGG	GTCTGAGGCGTGATTGCCT	146
*rpoC*	AACGAGCAGATGCCGAAGAA	AAATCGAGACCGTGACACCC	148
*sodC*(Cu/Zn)	TGACCTCACTTCGGTTCAGG	GGATGTTGCCGAAGTTGTCC	143
*sodA*(Fe/Mn)	TCGGTATCGTTCCGTTGCTC	GTGCAACGTCTTCCCAGTTG	123

#### 2.4.3 Relative gene expression analysis

The normalization of RT‐qPCR results should be performed with more than one validated reference gene (Bustin et al., 2009), and we used *gyrA*, *map, recA, rpoB, and rpoC* as some of the recommended bacterial reference genes (Rocha, Santos, & Pacheco, 2015). All reference genes had similar expression levels in the control and experimental groups except *recA*. The *recA* expression significantly varied between groups, and so we studied *recA* as the gene of interest. The other genes of interest were *CYP153*, *sodA*, and *sodC*. The PCR efficiency was determined with standard curve analysis and it counted 90%–100%. The relative levels of genes expression were calculated using ΔΔCt method (Bustin et al., 2009; Rao, Huang, Zhou, & Lin, 2013) taking into account PCR efficiency.

#### 2.4.4 Statistics

Data statistical analysis was conducted using R‐studio version 3.4.1 (https://www.rstudio.com/). The Shapiro–Wilk test was used to check the normality of the data. For data comparison, unpaired Student's *t*‐test and Mann–Whitney *U* test were utilized. Differences were considered statistically significant at *p* < 0.05.

## 3 RESULTS

### 3.1 *R. erythropolis* growth in the presence of hydrocarbons

**Figure A1:**
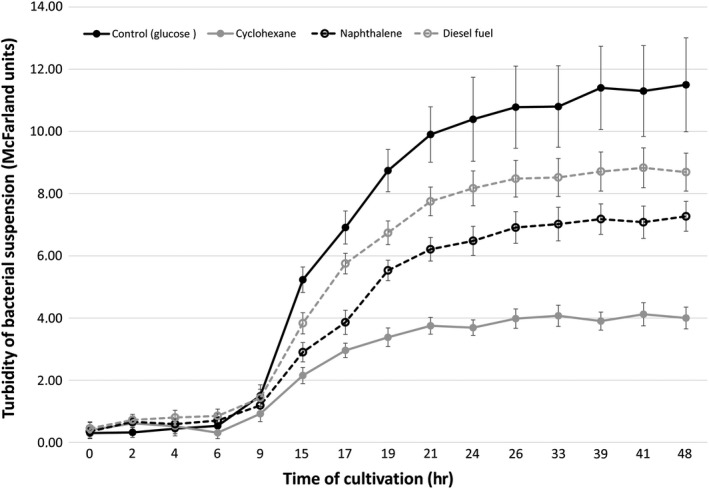
*R. erythropolis* growth curves with glucose and hydrocarbons (cyclohexane, naphthalene, and commercial diesel fuel). *R. erythropolis* was inoculated into 20 ml of basic mineral salt medium with the addition of 2% (400 μl) of hydrocarbons and incubated at 30°C with aeration. The average ± *SD* of three replicate cultures is shown

For culture of *R. erythropolis* grown in the basic mineral salt medium complemented of 0.5% of yeast extract with the addition of 2% w/v of the investigated hydrocarbons or glucose (as control), the growth curves were built (Appendix 1, Figure A1)**.** Analysis of the growth curves demonstrated that lag phase time was the same at cultivating *R. erythropolis* on different substrates. Similarly, the exponential phase time was the same for different substrates, as well as the stationary phase time. But *R. erythropolis* had the lowest increase in biomass (turbidity of suspension) when cultured on the medium with cyclohexane addition. The greatest turbidity of suspension was found in *R. erythropolis* cultured on the medium with the addition of glucose. After 20–22 hr of cultivation, the difference was threefold. Taken together, based on the similarity of time of various growth phases of *R. erythropolis* on different substrates, it can be assumed that bacterial cells mainly used the most complete substrate (0.5% yeast extract).

### 3.2 Superoxide generation by *R. erythropolis* in the presence of hydrocarbons

**Figure 1:**
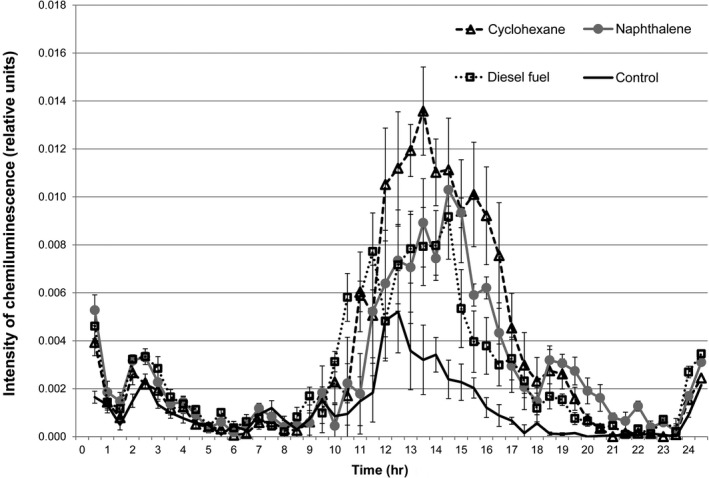
Intensity of superoxide anion radical generation (measured by lucigenin‐activated CL) upon incubation of *R. erythropolis* within 24 hr in basal mineral salt medium with the addition of hydrocarbons as a carbon source. Mineral salt medium with the addition of 1% of glucose was used as a control. Error bars are confidence interval limits. Differences between experimental and control measurements are statistically significant (*p* < 0.05) for all the data sets.

The data showing the influence of incubation time with hydrocarbons on lucigenin‐activated CL intensity of *R. erythropolis* are presented in Figure 1.

CL intensity was measured every 30 min during 24 hr. The maximal superoxide anion radical generation was registered between 12th and 17th hours when bacteria were incubated in the presence of hydrocarbons. Thereafter, the intensity of superoxide generation decreased again. Comparison of experimental and control groups make the differences in superoxide generation speeds obvious. The maximal, 2.8 times, CL stimulation of superoxide anion radical generation occurred after the addition of the cyclohexane. Incubation with the diesel fuel increased the generation speed by 1.8 times and doubled with the naphthalene.

### 3.3 Hydrogen peroxide generation by *R. erythropolis* in the presence of hydrocarbons

**Figure 2:**
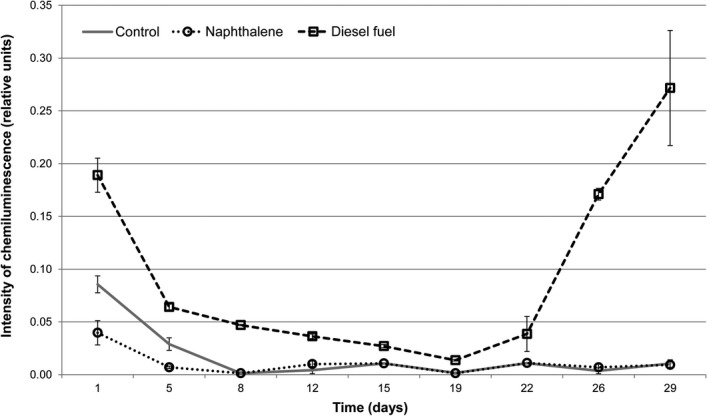
Intensity of hydrogen peroxide production (measured by luminol‐activated CL) upon incubation of *R. erythropolis* within 30 days in basal mineral salt medium with the addition of hydrocarbons as a carbon source. Mineral salt medium with the addition of 2% of glucose was used as a control. Error bars are confidence interval limits. Differences between experimental and control measurements are statistically significant (*p* < 0.05) for all the data sets.

Hydrogen peroxide accumulation in the culture medium was estimated according to the intensity of luminol‐activated CL in the presence of horseradish peroxidase. Measurements were taken for 30 days, and samples were taken every 3–4 days. As cyclohexane is highly volatile, it was not used in this long‐term experiment. H_2_O_2_measurements were performed only for microorganisms incubated with the diesel fuel and the naphthalene. The data showing the influence of hydrocarbons on peroxide accumulation are shown in Figure 2 **.**

Microorganisms incubated with the naphthalene produced 2.15 times less H_2_O_2_ in the culture medium than that in the control group. No significant differences were documented between the experimental and control groups starting from the eighth day of incubation in the presence of naphthalene. The production of the hydrogen peroxide by *R. erythropolis* incubated with the diesel fuel statistically exceeded the same in the control group throughout the experiment. On the first day, the H_2_O_2_ concentration in the experimental group was 2.19 times higher than in the control group and gradually decreased toward the middle of incubation. From 22nd day, the concentration of the peroxide increased and exceeded by 24.7 times the value in the control group by the end of incubation.

### 3.4 *R. erythropolis CYP153, recA, sodA,*and* sodC*gene expression in the presence of hydrocarbons

**Figure 3:**
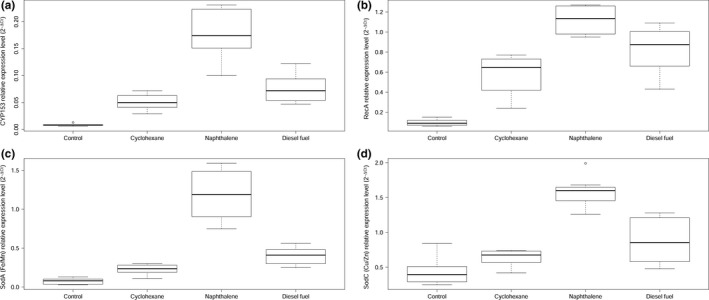
(a) *CYP153,* (b) *recA,* (c) Fe/Mn superoxide dismutase (*sodA*)*,* and (d) Cu/Zn superoxide dismutase (*sodC*) relative expression data (2^‐∆Ct^) upon incubation of *R. erythropolis* within 24 hr in basal mineral salt medium (control) and with the addition of hydrocarbons as a carbon source. Mineral salt medium with the addition of 2% of glucose was used as a control. Data are shown in typical box plot, displaying the minimum, first quartile, median, third quartile, maximum, and outliers. Differences between experimental and control measurements are statistically significant (*p* < 0.05) for all the data sets.

The data of investigated genes (*CYP153, recA, sodA, sodC)* relative expression (2‐∆Ct data) are presented in Figure 3.

First, it should be mentioned that for more clarity the gene relative expression levels were shown in Figure 3 according to 2‐∆Ct data, but for an accurate comparison, ∆∆Ct formula was used. Significant differences in *CYP153* expression was observed while comparing the control and experimental groups (Figure 3a). The media enriched by hydrocarbons increased *CYP153* transcription activity in bacteria: the addition of cyclohexane by 6 times, diesel fuel—8.2 times, and naphthalene increased *CYP153* mRNA level by about 20.7 times.

The *recA* transcription increased **(**Figure 3b) after incubation of bacteria with hydrocarbons: cyclohexane by 6.1, diesel fuel by 8.7, and naphthalene by 9.8 times, as it was in case of *CYP153* expression. It is important to note that *recA* expression is usually stable and it is often used for normalization of other genes (Rocha et al., 2015). In this study, we obtained quite opposite result.

The media enriched by hydrocarbons has also influenced superoxide dismutase encoding genes (*sodA, sodC*) expression (Figure 3c,d). The *sodA* expression was increased by 3.1 times after cyclohexane addition, 5.4 times with diesel fuel and most significant increment (16.1 times) was observed with extra naphthalene supplementation; whereas a statistically reliable increase (by 3.6 times) in *sodC* transcription, we found only in bacteria incubated in naphthalene‐enriched media.

## 4 DISCUSSION

Various manifestations of oxidative stress in the presence of polyaromatic hydrocarbons (PAHs), and, first of all, benz(a)pyrene (BAP), have been reported by different authors and for different taxonomic groups (Kang et al., 2007; Liu, Pan, Jin, & Cai, 2015; Tamburro et al., 2004; Weisman, Alkio, & Colón‐Carmona, 2010; Yang et al., 2014). Usually, only negative effects of PAHs including oxidative stress (Liu, Pan, et al., 2015; Weisman et al., 2010; Yang et al., 2014), imbalance of cellular metabolism and prevalence of catabolism over anabolic processes (Weisman et al., 2010), mutagenesis and cancerogenesis (Liu, Pan, et al., 2015; Yang et al., 2014), DNA damage (Yang et al., 2014) and cell death (Liu, Goa, et al., 2015; Weisman et al., 2010) are mentioned in the studies on multicellular organisms. We hypothesize that hydrocarbon‐induced oxidative stress can be both detrimental for individual bacterial cells but beneficial for adaptation of the bacterial population. ROS formed during oxidative stress can play a role in microbiological transformation of hydrocarbons and accelerate bacterial evolution intensifying mutagenesis and the genome recombination.

Initial stages of PAHs biotransformation (Kang et al., 2007; Lee, 1999; Tamburro et al., 2004), nitro derivatives of aromatic compounds (Pérez‐Pantoja et al., 2013), biphenyls, and their chlorinated derivatives (PCBs) (Ponce et al., 2011) cause oxidative stress in bacterial cells. The same happens in process of microbial utilization of alkanes (Sazykin et al., 2016, 2018). Both products of PAHs partial oxidation—hydroquinones (Shimada, 2006) and suboptimal substrates of enzymes of hydrocarbons oxidation initial stages (Pérez‐Pantoja et al., 2013) lead to ROS generation. Hydroquinones undergo redox cycling with the production of O_2_^·−^, and on the other hand, enzymatic cycle with suboptimal substrates often ends up in shunting and ROS production.

Bacterial cytochromes can act as ROS generating enzymes. They are involved in the synthesis of secondary metabolites and utilization of hydrophobic substrates, such as hydrocarbons. Many bacterial monooxygenases, involved in the initial stages of hydrocarbons oxidation, belong to P450 family cytochromes (Khmelevtsova, Sazykin, Sazykina, & Seliverstova, 2017). P450 family monooxygenases of are present in many hydrocarbon‐degrading microorganisms (van Beilen & Funhoff, 2007; Bowman & Deming, 2014; Kubota et al., 2005; Liu, Gao, et al., 2015). Generally, cytochromes P450 involved in oxidation of hydrocarbons belong to alkane hydroxylases (van Beilen et al., 2006; Funhoff, Bauer, Garcia‐Rubio, Witholt, & Beilen, 2006; Maier, Förster, Asperger, & Hahn, 2001; Rojo, 2009). Such enzymes were discovered also in *Rhodococcus*. Other studies have shown that cytochromes can oxidize both linear alkanes and aromatic hydrocarbons (Bell & Wong, 2007; Du et al., 2006; Ignatovets, Akhramovich, & Leontiev, 2009).

It is well known that in the course of enzymatic reactions of cytochrome P450 the so‐called “disjunction” of the cycle may occur when the flow of electrons derived from NAD(P)H to P450 molecules leads to the generation of superoxide anion radical and/or hydrogen peroxide instead of the products of monooxygenase reaction (Guengerich, 2001; Goeptar, Scheerens, & Vermeulen, 1995).

In this study, we demonstrated the increase of *CYP153* transcription in *R. erythropolis* cultivated with hydrocarbons. The naphthalene addition caused the greatest induction (20.7‐fold). Cyclohexane and diesel fuel caused a weaker effect—6.0‐ and 8.2‐fold, respectively.

Since bacterial P450 cytochromes could be a source of superoxide anion radicals, we observed the simultaneous induction of *sodA* and *CYP153* genes. Cyclohexane increased the transcription of *CYP153* by 6 times and *sodA* by 3.1 times, diesel fuel—8.2 and 5.4 times, and naphthalene—20.7 and 16.1 times, respectively. Besides, only naphthalene introduction led to upregulation of the* sodC* expression.

The present data obtained for *R. erythropolis* coincided with our previous results of reactive oxygen species production by *Acinetobacter calcoaceticus* (Sazykin et al., 2016) and *Achromobacter xylosoxidans* (Sazykin et al., 2018). Namely, O_2_^·−^ production increased during first 12 hr of bacteria incubation with hydrocarbons, and then it decreased. However, microorganisms of different bacterial taxa differ in ROS generation depending on hydrocarbons type. In *R. erythropolis* and *A. xylosoxidans* (Sazykin et al., 2018), the maximum production of O_2_^·−^ was caused by cyclohexane, and in *A. calcoaceticus*—by diesel fuel and PAHs (Sazykin et al., 2018). Most likely, it is associated with great importance of O_2_^·−^ production in a prokaryotic cell when shunting an enzymatic cycle by suboptimum substrates, and, respectively, presence of hydrocarbons oxidases with different specificities in various microorganisms. In prokaryotes, the contribution of ROS caused by oxidized PAHs derivatives is much lower compared to the eukaryotic cell, possibly, due to the fact that PAHs immediately disrupt electron transport chain in the eukaryotic cell.

Decrease in O_2_^·−^ in the final stage of the 24‐hr incubation, presumably, is not due to the reduction of superoxide production in a cell, but due to the increase of the bacterial superoxide dismutase (SOD) expression, mainly *sodA*, but not *sodC*. The transcription activity of Fe/Mn SOD (encoded by *sodA*) in *R. erythropolis* incubated with various hydrocarbons increased from 3.1 (cyclohexane) to 16.1 (naphthalene) times. At the same time, expression of *sodC* increased by 3.6 times only in the presence of naphthalene, but not in the presence of cyclohexane and diesel fuel.

Higher *sodA* in comparison to *sodC* expression is important for bacterial cell protection against O_2_^·−^ radicals as it was shown for *P. aeruginosa* (Hassett, Schweizer, & Ohman, 1995). Significant increase in SOD enzymatic activity caused by hydrocarbons was demonstrated for such bacteria as *A. calcoaceticus* and *A. xylosoxidans* earlier (Sazykin et al., 2016, 2018). In this case, hydrocarbon degraders are protected from the reactive type of ROS—superoxide anion radical generated at the initial stages of hydrocarbons oxidation, transforming it into much stable form—hydrogen peroxide.

H_2_O_2_formed in bacteria is transported outside the cell and accumulates in the environment. There are different hydrogen peroxide enrichment patterns of culture medium for hydrocarbon‐degrading bacteria incubated with various hydrocarbons. Accumulation of H_2_O_2_ in *R. erythropolis* in the presence of diesel fuel, but not naphthalene has been demonstrated. Hydrocarbons have a similar effect on *A. calcoaceticus* and *A. xylosoxidans*. Besides, reduction of catalase activity has been registered in such microorganisms as *Gordona terrae, Rhodococcus rubropertinctus, R. erythropolis, A. calcoaceticus,* and *A. xylosoxidans* in the course of hydrocarbons biotransformation (Gogoleva, Nemtseva, & Bukharin, 2012; Sazykin et al., 2016, 2018). The reduction of catalase activity promotes H_2_O_2_accumulation and organic substrates oxidation by ROS in cells environment. In the case of hydrophobic substrates, for example, hydrocarbons, oxidation facilitates their higher bioavailability due to the hydrophobic properties weakening and formation of surface‐active substances.

The increased expression of* recA* gene in *R. erythropolis recA* allows us to propose that the oxidative stress induced by hydrocarbons leads to DNA lesions. It is notable, that accumulation of *recA* transcript has also coincided with increase in *CYP153* gene expression. The ROS produced by cytochrome P450 are likely to cause DNA damage. In turn, the stimulated SOS response increases mutagenesis in bacterial population, and probably intensifies horizontal gene transfer. Increase in ROS generation as a result of oxidation of suboptimal substrate, 2,4‐dinitrotoluene (DNT) xenobiotic, is described for *Burkholderia sp*. DNT. Consecutively, the oxidative stress led to DNA damage with the formation of 8‐hydroxy‐2'‐deoxyguanosine (8‐oxoG) and increased mutagenesis. In this way, oxidative stress may lead to enzyme evolution acceleration and bacterial adaptation to new substrates and ecological niches (Pérez‐Pantoja et al., 2013).

Two recent works support this proposal (Akkaya, Nikel, Pérez‐Pantoja, & Lorenzo, 2019; Akkaya, Pérez‐Pantoja, Callesc, Nikel, & Lorenzo, 2018). The genetic cluster encoding the metabolic pathway of the DNT oxidation in *Burkholderia cepacia* R34 was transferred to *Pseudomonas putida* (Akkaya et al., 2018). When DNT is oxidized in a bacterial cell, the level of ROS increases tenfold and the level of homologous recombination increases fourfold. Activation of the SOS response (induction of the promoter of the *recA* gene) and influence of ROS on the mutagenesis level were not registered. The authors believe that due to the high level of redox metabolism, *Pseudomonas putida* eliminates the negative effects of oxidative stress. However, it remains unclear how the efficiency of the homologous recombination increases without activation of SOS response and how the evolution of enzyme systems and genetic plasticity is accelerated without increasing the level of mutagenesis.

The same metabolic pathway gene cluster from *Burkholderia cepacia* R 34 was moved to *Escherichia coli* (Akkaya et al., 2019). DNT degradation in *E. coli* lead to increased mutagenesis, and direct damage to DNA. However, the *recA* gene promoter was not activated. The authors concluded that the increase in mutagenesis was not due to direct DNA damage and SOS response, but due to the stress‐induced decrease of DNA replication accuracy.

The data obtained for bacterial species support the idea that ROS can accelerate molecular evolution by different mechanisms in various microorganisms. For example, in *Burkholderia*, the main contribution was by direct DNA damage, in *Rhodococcus*—by SOS response, in *E. coli*—it was by decrease in accuracy of DNA replication. These processes do not cause only damages to cells but represent a mechanism of procrustean adaptation to changing nutritional conditions of the environment.

In case of *Pseudomonas putida*, high level of intracellular antioxidants protects bacterial cells from negative effects of the oxidative stress, but it might also reduce the rate of changes in the cell's enzyme systems and its adaptability to new substrates. Taken together, the antioxidant systems possibly determine plasticity and diversification of bacterial population. The balance between bacterial cells survival and the ability of microorganisms to colonize new ecological niches is still an extremely intriguing problem.

## 5 CONCLUSIONS

Addition of cyclohexane, diesel fuel, or naphthalene increases the *CYP153* gene expression and production of superoxide anion radical in hydrocarbon‐degrading *R. erythropolis*. The expression of Fe/Mn superoxide dismutase (*sodA*) and *recA* genes proliferates also. Further, the quantity of *sodC* (Cu–Zn superoxide dismutase) mRNA elevates in the presence of naphthalene. Bacteria incubated with diesel fuel accumulate hydrogen peroxide in the culture medium. Therefore, biotransformation of such hydrocarbons as alkanes, cycloalkanes, and aromatic hydrocarbons leads to oxidative stress and intensifies enzymatic antioxidant protection and DNA reparation in *R. erythropolis* cells.

## CONFLICT OF INTERESTS

The authors declare that they have no conflict of interest.

## AUTHOR CONTRIBUTIONS

I.S. and M.S. designed the study. M.M., L.K., and E.S. collected and analyzed the data. I.S., M.M., A.R., and M.S. wrote the manuscript. All authors read and contributed to the manuscript.

## ETHICS STATEMENT

None required.

6

## Data Availability

The 16S rRNA genes sequence of *Rhodococcus erythropolis* strain was deposited in the GenBank database under accession number https://www.ncbi.nlm.nih.gov/nuccore/MH718753.
